# Heparin-induced thrombocytopenia during IgA vasculitis: a case report

**DOI:** 10.1093/omcr/omab002

**Published:** 2021-03-08

**Authors:** Kaisei Yamasaki, Moe Kyotani, Yasuyo Urase, Yoichi Goto, Tsuneaki Kenzaka

**Affiliations:** 1 Department of internal medicine, Yoka Municipal Hospital, Yabu, Hyogo, Japan; 2 Division of Community Medicine and Career Development, Kobe University Graduate School of Medicine, Kobe, Hyogo, Japan

**Keywords:** immunoglobulin A vasculitis, heparin-induced thrombocytopenia, hemodialysis, palpable purpura, edema

## Abstract

Immunoglobulin A (IgA) vasculitis is characterized by small vessel vasculitis involving immune complexes and IgA deposition. The development of heparin-induced thrombocytopenia (HIT) during IgA vasculitis is extremely rare. An 87-year-old man presented with general fatigue, leg edema, purpura, arthritis and renal disease. He was diagnosed with IgA vasculitis and was admitted to our hospital. Hemodialysis with heparin was initiated thrice a week on post-admission Day 11. On Day 21, during hemodialysis, the pressure in the dialysis circuit increased and the dialysis was interrupted. On Day 24, the platelet count rapidly decreased to 18 000/μl. The patient was diagnosed with HIT after testing positive for HIT antibodies; heparin was discontinued at this time. Precautions must be taken against the onset of HIT when initiating hemodialysis in patients with IgA vasculitis.

## INTRODUCTION

Immunoglobulin A (IgA) vasculitis is characterized by small vessel vasculitis involving immune complexes and IgA deposition [1]. Diagnosis is based on the 2010 European League Against Rheumatism (EULAR)/Paediatric Rheumatology International Trials Organisation (PRINTO)/Paediatric Rheumatology European Society (PRES) [2] or the 1990 American College of Rheumatology (ACR) classification criteria [3], which are commonly used in Japan.

Heparin-induced thrombocytopenia (HIT), a rare complication of heparin therapy, activates platelets, inducing thrombocytopenia and a prothrombotic state [4]. The 4Ts score is required for clinical diagnosis; a definitive diagnosis can only be made if the serum anti-HIT antibodies are detected [4]. During dialysis introduction, HIT is found in 3.9% of cases [4].

Our patient exhibited acute kidney failure owing to IgA vasculitis and HIT on initiating dialysis.

## CASE PRESENTATION

An 87-year-old man was being treated for chronic kidney disease at a local clinic; he was referred to our hospital because of general fatigue and leg edema and was admitted.

**Figure 1:**
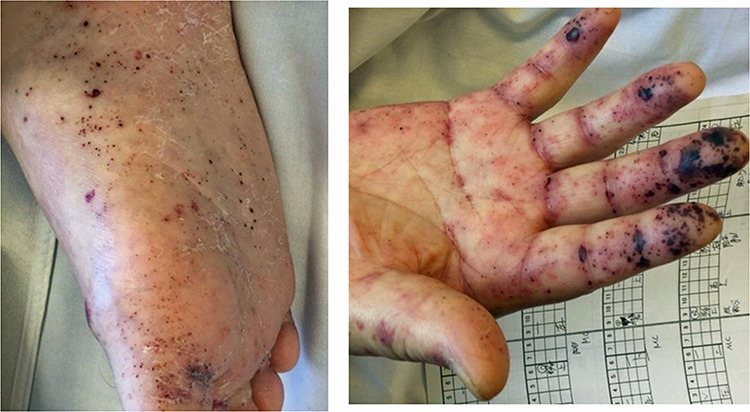
Skin findings on Day 10; worsening of purpura was observed on both the hands, lower legs, soles and dorsum pedis.

Physical examination at admission showed a clear consciousness, blood pressure: 203/93 mmHg, body temperature: 36.0°C and SpO2: 98% on room air. Mild pitting edema was observed on both lower legs. The serum creatinine level (normally around 1.1 mg/dl), 4.4 mg/dl on the day of hospitalization; blood and urine test results at admission are shown in Table 1. Arthritis of the right hand was observed on admission. Palpable purpura on both upper and lower limbs was observed from Day 4, and spontaneous pain and tenderness of the entire abdomen was observed from Day 5. Creatinine levels increased from 6.09 mg/dl on Day 8 to 10.1 mg/dl on Day 10, and blood clots were observed in the stool before dialysis. On Day 10, the patient presented with purpura (Fig. 1), arthritis and renal involvement and was diagnosed with IgA vasculitis.

**Table 1:** Blood and urine tests on Day 1

Test parameters	Laboratory test value	Normal range
White blood cells	14 000/μl	3000–8300/μl
Neutrophils	84%	41–74%
Lymphocytes	5%	18–48%
Eosinophils	4%	0–5%
Hemoglobin	11.7 g/dl	13.5–17.5 g/dl
Mean corpuscular volume	77.3/fl	85–102/fl
Platelets	246 × 103/μl	130–330 × 103/μl
Prothrombin time/International normalized ratio	1.08	0.85–1.15
Activated partial thromboplastin time	25.7 s	24.3–36.0 s
Fibrinogen	232 mg/dl	150–400 mg/dl
Fibrin degradation product	15.1 μg/ml	0–9.9 μg/ml
D-dimer	6.7 μg/ml	0–0.99 μg/ml
Total protein	5.2 g/dl	6–8.4 g/dl
Albumin	2.4 g/dl	3.1–5.5 g/dl
Lactate dehydrogenase	251 U/l	106-211I U/l
Creatine phosphokinase	74 U/l	30-180I U/l
Blood nitrogen urea	50.5 mg/dl	6.2–19.4 mg/dl
Creatinine	4.45 mg/dl	0.8–1.2 mg/dl
Estimated glomerular filtration rate	10.5 ml/min/1.73 m2	
Sodium	123 mEq/l	136–148 mEq/l
Potassium	3.0 mEq/l	3.6–5 mEq/l
C-reactive protein	1.03 mg/dl	0–0.6 mg/d
Urine specific gravity	1.007	1.002–1.03
Urinary protein	3+	
Occult blood in urine	3+	
Leukocytes in urine	Negative	
Bacteria	Negative	
Granular casts	30–49/full field	
Hyaline casts	50–99/full field	
Waxy casts	+/full field	
Urinary protein	14.57 g/g Cr	

Results of blood tests performed for differential diagnosis are shown in Table 2.

**Table 2:** Additional blood tests on Day 4

Test parameters	Laboratory test value	Normal range
Anti-streptolysin O	5 IU/ml	0–240/IU/ml
Anti-streptokinase antibody	1:5	1:0–99 999
Syphilis rapid plasma reagin	Negative	
Quantitative treponema pallidum hemagglutination	80%	0–79.9%
Antinuclear antibodies	1:40	1:0–39.9
SS-A antibodies	1.0 U/ml	0.0–9.99 U/ml
SS-B antibodies	1.0 U/ml	0.0–9.99 U/ml
Proteinase 3 antineutrophil cytoplasmic antibodies	1.0 U/ml	0–3.49 U/ml
Myeloperoxidase–ANCA	1.0 U/ml	0–3.49 U/ml
Anti-glomerular basement membrane antibodies	2.0 U/ml	0–2.99 U/ml
IgA antibodies	223 mg/dl	80–450 mg/dl
IgG antibodies	446 mg/dl	800–1800 mg/dl
IgM antibodies	98 mg/dl	60–280 mg/dl
Hepatitis B surface antigens	0.3 COI	0–0.9 COI
HBS antibodies	0.2 mIU/ml	0–9.9 mIU/ml
Hepatitis C virus antibodies	0.1 COI	0–0.9 COI
Soluble IL2 receptor antibodies	1950 U/ml	145–519 U/m
Coagulation factor XIII activity	34%	70–140%

Ig, immunoglobulin; HBS, hepatitis B surface.

**Figure 2:**
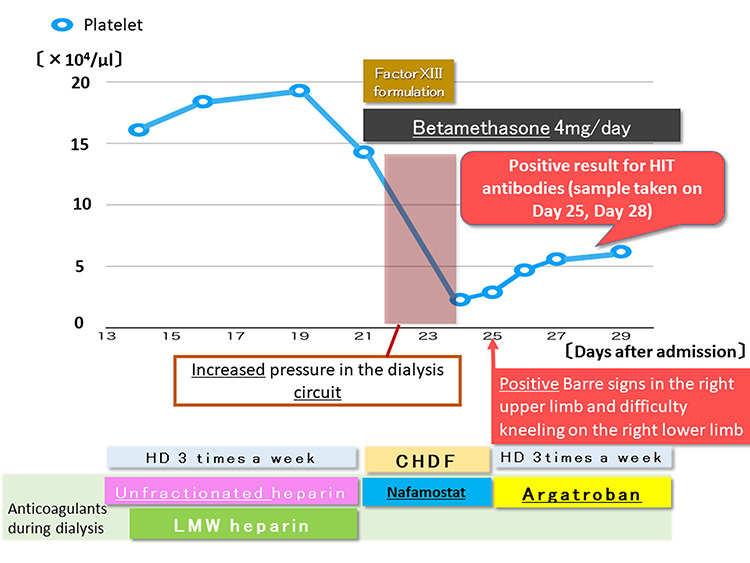
Platelet count over time from Day 13 and treatment course.

Regarding the treatment of IgA vasculitis, the patient and his family were advised on the need for treatment with steroids; however, they did not agree because of the susceptibility to infection. Hemodialysis (HD) was administered thrice weekly since Day 11 (Fig. 2). Until Day 13, unfractionated heparin was administered both as an anticoagulant in the circuit and for flushing the route. From Day 14, the anticoagulant in the circuit was switched to low-molecular-weight heparin (LMW), and unfractionated heparin was used to flush the route. On Day 21, the pressure in the dialysis circuit increased during hemodialysis, causing interruption. The patient was switched to continuous hemodiafiltration (CHDF) on the same day owing to worsening symptoms of congestion caused by inadequate hydration. On the same day, the patient and his family were advised on the need for treatment with steroids; they provided consent, and betamethasone was started at a dose of 4 mg/day. The abdominal pain worsened; this was considered to be an abdominal symptom related to IgA vasculitis, and blood coagulation factor XIII was therefore administered. Furthermore, as the platelet count was 143 000/μl, the anticoagulant in the circuit was changed to nafamostat owing to gastrointestinal bleeding. On Day 24, the platelet count rapidly decreased to 18 000/μl. The 4Ts score [2] was five points and heparin was discontinued. On Day 25, positive Barre signs in the right upper limb and difficulty in kneeling on the right lower limb were observed; thus, cerebral thrombotic symptoms of HIT were suspected and argatroban was initiated. This improved the dialysis circuit and stabilized the intracircuit pressure; therefore, the patient was switched back from CHDF to hemodialysis.

On Day 26, the paralytic symptoms disappeared; on Day 28, the patient tested positive for HIT antibodies (sample taken on Day 25), confirming the diagnosis of HIT, and on Day 34, the platelet count improved to 95 000/μl. Betamethasone did not improve the renal function and resulted in steroid psychosis. Therefore, the patient’s ability to provide informed consent for dialysis was lost, resulting in difficulties in performing the procedure. Eventually, on Day 59, he died of respiratory failure due to renal failure.

## DISCUSSION

To the best of our knowledge, this is the first report describing the onset of HIT during the management of IgA vasculitis. The patient was clinically diagnosed with IgA vasculitis. Skin and kidney biopsies were not performed because the patient did not provide consent. However, cholesterol crystal embolism was unlikely, as macrovascular surgery, catheterization and anticoagulant therapy had not been performed prior to the appearance of purpura. Regarding disseminated intravascular coagulation (DIC), the acute DIC score was three points (platelets only), which was negative; complement levels were not measured. Therefore, possible causes of palpable purpura were ruled out, and the 2010 EULAR/PRINTO/PRES [2] (purpura plus arthritis and renal involvement) and ACR classification criteria [3] (palpable purpura and intestinal angina) were met; IgA vasculitis was diagnosed.

The patient had been referred to use for chronic kidney disease; however, we were not able to investigate the etiology.

Abdominal symptoms may have been caused by thrombosis rather than by IgA vasculitis; however, abdominal imaging findings were only obtained using plain computed tomography (CT) throughout the course. It is unlikely that HIT was involved before the appearance of central nervous system (CNS) symptoms. Abdominal symptoms were present since admission, and they gradually worsened, suggesting that it was unlikely for the abdominal symptoms to be related to thrombosis.

The hemodialysis membrane used for intermittent hemodialysis was NV-10 U (Toray Medical Co, Ltd), and that for CHDF was AEF-07 (ASAHI KASEI MEDICAL CO, LTD). Both have been reported to cause thrombocytopenia, although the frequency is unknown; however, no CNS symptoms have been reported. Accumulation of further cases is desirable.

The issue of deformation of urinary red blood cells was unknown as no tests had been performed.

Since the 4Ts score of the patient was five points based on thrombocytopenia over 50%, a significant decrease in platelet count 10 days after heparin administration and a lack of other causes of thrombocytopenia, HIT could not be excluded [5]. HIT was diagnosed based on the 4Ts score and confirmed antibody positivity. HD circuit clots of unknown origin are the initial abnormality in HIT [6, 7]. In this patient, pressure in the dialysis circuit increased from Day 21, and platelet counts decreased from 193 000 to 143 000/μl (Fig. 2); this is abnormal in early stages of HIT. The onset of HIT is typically within 5–10 days [5]. In the present case, the onset was on the 10th day after initiating heparin administration. The transiently occurring neurological symptoms (positive Barre signs in the right upper limb and difficulty kneeling on the right lower limb) may have been caused by HIT. Symptoms resolved 2 days after discontinuing heparin and 1 day after initiating argatroban. Intermittent use of heparin for dialysis may have led to mild symptoms. Cranial imaging was not feasible as he could not be transported.

Pathologically, IgA vasculitis is an immune-related leucocytoclastic vasculitis caused by IgA immune complexes with specific fibrin deposition in the vessel walls visible on immunofluorescence [8]. It is unclear how IgA affects the pathogenesis of HIT. Reports of antineutrophil cytoplasmic antibody (ANCA)-associated vasculitis causing HIT are rare [9, 10]; it is presumed to increase the co-morbidity of other autoimmune diseases and autoantibody production.

No previous reports exist of a direct association between ANCA-associated vasculitis and HIT antibody production. The association between IgA vasculitis and HIT is unknown and may have occurred incidentally while initiating hemodialysis. However, IgA vasculitis is presumably a systemic vasculitis, involving immune complexes, and easily causes antibody production; this may have affected the HIT antibody production. Therefore, the occurrence of HIT should be considered in IgA vasculitis at the time of initiating dialysis.
